# Effects of Cholinergic and Opioid Antagonists on In Vitro Release of Met-Enkephalin, Somatostatin and Insulin-like Growth Factor-1 by and PENK Expression in Crop, Proventriculus and Duodenum of Newly Hatched Chickens

**DOI:** 10.3390/ani15121702

**Published:** 2025-06-09

**Authors:** Colin G. Scanes, Klaudia Jaszcza, Alina Gajewska, Krystyna Pierzchala-Koziec

**Affiliations:** 1 Department of Biological Sciences, University of Wisconsin Milwaukee, 3209 N Maryland Ave, Milwaukee, WI 53211, USA; 2 Department of Animal Physiology and Endocrinology, University of Agriculture in Krakow, Mickiewicza 21, 31-120 Krakow, Poland; klaudia.jaszcza@onet.pl (K.J.); rzkoziec@cyf-kr.edu.pl (K.P.-K.); 3 The Kielanowski Institute of Animal Physiology and Nutrition, Polish Academy of Sciences, Instytucka 3 Street, 05-110 Jabłonna, Poland; a.gajewska@ifzz.pl

**Keywords:** met-enkephalin, somatostatin (SRIF), insulin-like growth factor-1, crop, proventriculus, duodenum, chicken, cholinergic, opioid

## Abstract

**Simple Summary:**

It is increasingly recognized that the functioning of the gastrointestinal (GI) tract is controlled by nerves, peptides and proteins released from cells in different regions of the foregut. The present study examines aspects of those controls using GI tissue from young chicks incubated in vitro. Three hormones/peptides were found to be released from the GI tract with their release controlled by specific types of nerves. Two GI hormones/peptides increased cell division and, consequently, the growth of the GI tract.

**Abstract:**

The gastrointestinal (GI) tract is under neural, endocrine and paracrine control. The release (basal and in the presence of either cholinergic and opioid antagonists) of Met-enkephalin, insulin-like growth factor 1 (IGF-1) and somatostatin (SRIF) was determined quantitatively in vitro using explants of the crop, proventriculus and duodenum from either day 0 or day 1 chicks. In addition, the effects of cholinergic and opioid antagonists on PENK gene expression were examined. Thus, the aim of this study was to determine the roles of cholinergic and opioid receptors in the GI tract in newly hatched chickens. Moreover, the effect of IGF-1 and Met-enkephalin on cell division in duodenal explants in vitro was determined. There was both the release of Met-enkephalin from, and PENK expression in, the explants of the crop, proventriculus and duodenum tissue. This is the first report of any neuropeptide(s) being synthesized in and/or released from the crop. In vitro release of Met-enkephalin, IGF-1 and SRIF from duodenal and proventriculus explants was influenced (*p* < 0.01) by either cholinergic or opioid antagonists; for instance, in the presence of atropine, decreases (*p* < 0.001) of 17.8% and 57.7% are seen, respectively, in Met-enkephalin release and PENK expression in crop explants from day 1 chicks. Moreover, in the presence of atropine, there were increases (*p* < 0.001) of 47.7% and 70.9% in IGF-1 release in proventriculus explants from, respectively, day 0 and day 1 chicks. Met-enkephalin and/or IGF-1 stimulated the cell division of duodenal explants in vitro. This is the first report of Met-enkephalin release and PENK expression in the avian crop and of the effects of cholinergic or opioid antagonists on these factors. It is also the first report of either cholinergic or opioid control of IGF-1 release in the periphery of any species. There were strong relationships (*p* < 0.05) between the release of Met-enkephalin and that of IGF-1 in the duodenum and between the release of SRIF and that of IGF-1 in the proventriculus. This is the first report of IGF-1 and Met-enkephalin stimulating (*p* < 0.001) the proliferation of duodenal cells and, together, exerting a synergist effect. It is concluded that the release of Met-enkephalin, IGF-1 and SRIF from foregut regions is under tonic cholinergic and opioid control.

## 1. Introduction

There is increasing interest into the physiological control of gastro-intestinal (GI) function and particularly the role of neuropeptides. The present communication predominantly focuses on one neuropeptide, namely Met-enkephalin. This is found in both neurons (myenteric ganglia and myenteric plexus) and enteroendocrine cells in the small intestine and stomach of mammals including humans [1,2], guinea pigs [3], cats [4], dogs [5], mice [6], rat and pigs [7]. Moreover, Met-enkephalin has been reported in the submucosal plexus of the proventriculus and duodenum in chick embryos [8]. In birds, the crop is a sac-like structure connected to and derived from the esophagus (reviewed: [9]). As such, it was viewed as unlikely to contain neuropeptides. What is not known is whether the crop, proventiculus and duodenum in birds synthesize and release Met-enkephalin.

There is relatively little published data on the control of Met-enkephalin release and synthesis in the GI tract. The release of Met-enkephalin is shifted during physiological changes in the GI tract such as during peristalsis [10,11] or following a meal [12]. There is evidence, at least, for cholinergic agonists influencing Met-enkephalin release and/or synthesis in multiple systems in mammals. For instance, the cholinergic/nicotinic agonist, nicotine, stimulated the release of Met-enkephalin from bovine chromaffin cells [13]. Similarly, the effects of the nicotine on Met- enkephalin in the jejunum have been reported [14]. In rats, the administration of either the muscarinic antagonist, atropine, or the nicotinic antagonist, hexamethonium, was followed by increased expression of proenkephalin (PENK) [15]. In contrast, in vitro release of Met-enkephalin was decreased in the presence of either atropine or hexamethonium [15].

The availability of media from explants incubated with or without cholinergic or opioid antagonists allowed for the determination of the effects on the release of somatostatin (SRIF) and insulin-like growth factor-1 (IGF-1) in birds. Both hormones play important roles in GI functioning (discussed below). Neither SRIF nor IGF-1 was detected in media incubated with crop explants. IGF-1 is found in the GI tract throughout the chordates including mammals such as neonatal cattle [16] and camels [17] and even non-vertebrate chordates including *Ciona intestinalis* and *Branchiostoma lanceolatum* [18,19]. Exogenous IGF-1 stimulates the proliferation of small intestine epithelial cells [20,21,22]. It was hypothesized that IGF-1 would be released from the proventriculus and duodenum explants and stimulate cell proliferation in the small intestine. It was further hypothesized that the rate of release of IGF-1 would not be influenced by the presence of cholinergic antagonists, as there are no published reports of the cholinergic effect on IGF-1 release in the periphery or of neural control of IGF-1 release from GI cells [23].

Immuno-reactive SRIF has been shown to be present in both the proventriculus and small intestine of chickens [24]. Based on the ability of the cholinergic antagonist, atropine, to influence the release of SRIF from the stomach [25,26,27], there would appear to be ubiquitous muscarinic (cholinergic) control of SRIF secretion. What is not known is whether there are nicotinic effects or whether SRIF secretion from the small intestine is under cholinergic control. It is noted that SRIF in present in both D enteroendocrine cells and in the enteric nervous system (pigs: [28]; also reviewed: [29,30]). Moreover, nicotinic receptors have been reported to mediate, at least partially, the cholinergic anti-inflammatory effect [31].

Met-enkephalin has been termed the opioid growth factor [32,33,34,35,36,37,38,39,40,41], with Met-enkephalin decreasing cell proliferation in astrocytes [32,42]; aortal endothelial, smooth muscle and fibroblast cells [33]; corneal epithelial cells [38]; epidermal cells [35]; esophageal cells [34]; T and B lymphocytes [36,37]; and cancer cells [39,40,41]. In contrast, retinal pigment epithelial cell growth was stimulated by Met-enkephalin [43]. What is not known is whether Met-enkephalin influences the cell proliferation of gastro-intestinal cells.

The primary aims of this study were to determine whether the crop, proventriculus and duodenum of young chickens release Met-enkephalin in vitro and/or express proenkephalin (PENK) and whether both are under cholineric and/or opioid control. These aims were examined using cholinergic and opioid antagonists, namely the muscarinic antagonist, atropine; the nicotinic antagonist, hexamethonium; and the μ-opioid antagonist, naltrexone. A secondary aim of this study examined the effect of cholinergic antagonists and an opioid antagonist on the in vitro release of SRIF and IGF-1 from the proventriculus and duodenum of chicks. A third aim was to determine whether Met-enkephalin, in the presence or absence of IGF-1, influences duodenal cell proliferation in newly hatched chicks.

## 2. Materials and Methods

Eggs and their Incubation: Hatching eggs [egg weight—mean 60.3 ± SEM 1.11 g] of the Ross 308 broiler chicken parental line (Aviagen) were obtained from a commercial farm in Poland. The eggs were incubated in a Brinsea-type OVA-Easy advance incubator (Weston-super-Mare, North Somerset, UK) under standard conditions, i.e., a temperature of 37.8 ± 0.1 °C and a relative humidity (RH) value of 50% ± 2%. Immediately after hatching (day 0) or 24 h later (day 1), chicks were transported to the laboratory and euthanized within 2 h by cervical dislocation.

Ethical approval: The experiments on chickens involved euthanizing animals solely for the use of their organs or tissues. Consequently, the in vitro studies performed did not require the consent of the Local Ethics Committee, in accordance with Article 2.1 from the Act of Law of 15 January 2015 (Journal of Laws of 2015, item 266), on the protection of animals used for scientific or educational purposes.

Animals: The rationale for employing 0- and 1-day-old chicks without access to feed was to negate any confounding effects of ingesta on GI functioning. The study was carried out in two phases: phase 1 employed tissues from chicks within 2 h of hatching (stated as day 0), and phase 2 used tissue from one-day-old chicks (stated as day 1).

Experimentation: In both phases, tissue explants from 25 newly hatched chicks were divided into 5 treatment groups and were incubated in vitro without antagonists (control, 0), with the muscarinic antagonist (cholinergic) atropine (At, 100 nM) and/or the nicotinic antagonist (cholinergic) hexamethonium (Hx, 100 nM) or with the μ opioid antagonist naltrexone (Nal, 100 nM). The design allowed for the analysis of 2-way ANOVA of the effects of cholinergic antagonists in each of the two phases separately. The same control chicks were employed for examining the effects of opioid antagonists to reduce the number of animals employed in experimentation.

Tissue culture: Explants of the crop, duodenum and proventriculus (each fragment 50–70 mg) were dissected and placed on a 24-well plate (tissue from each chick in a separate well). Tissues were incubated in 1 mL of Eagle’s medium supplemented with 0.05% bovine serum albumin and 2 µL of an antibiotic–antimycotic solution (*n* = 5) for 6 h at 38 °C (5% CO_2_) in Eagle’s medium in the presence or absence of receptor antagonists. Following incubation, the tissues were placed in StayRNA (A&A Biotechnology, Gdynia, Poland) until RNA isolation. The culture media were stored at −80 °C prior to determination of Met-enkephalin, SRIF and IGF-1.

Concentrations of hormones: Met-enkephalin concentrations were determined via radioimmunoassay [44]. IGF-1 and SRIF concentrations in culture media were determined using RIA kits (RIA-4702, RIA-3017, DRG, Marburg, Germany).

Gene Expression Analysis (RNA Isolation, Reverse Transcription Reaction and qPCRReaction): RNA was isolated with the TRI Reagent according to the method by Chomczynski and Sacchi [45]. The quality and concentration of the isolated RNA were determined via spectrophotometric analysis at wavelengths of 260 and 280 nm. Reverse transcription reactions were performed in accordance with the manufacturer’s recommendation using the primers shown in Table 1. The reaction mixture contained 4.2 µL of sterile water, 2 µL of 10 *×* RT buffer, 0.8 µL of 25 *×* dNTP MIX (100 nM), 2 µL of the 10 *×* RT primer (random primer), 1 µL of MultiScribeTM reverse transcriptase, 2 µg of total RNA in 10 µL of water. Reverse transcription reactions were performed in a thermocycler (Personal Thermal cycler, Eppendorf, Hamburg, Germany) with the following cycle settings: 25 °C—10 min, 37 °C—120 min and 85 °C—5 min. The obtained cDNA constituted a template for the qPCR reaction, and it was stored at −20 °C. qPCR reactions were performed in a 96-well thermal cycler (StepOne Plus, Applied Biosystems, Foster City, CA, USA). The 18S rRNA gene was used as a reference gene (Table 1). The following program was used: 15 min at 95 °C, followed by 40 cycles of 15 s at 95 °C, 20 s at 62 °C and 20 s at 72 °C for the 10 µL reaction mixture containing 2 µL of 5 *×* Hot FIREPol Eva Green qPCR, 0.12 µL of primers (10 pmol/µL) and 1 µL of cDNA (10-fold diluted sample from the RT reaction). A duplicate experiment was performed for each sample. Expression was calculated using the 2^−ΔΔCt^ method. The StepOne program was used for quantification. Expression was calculated using the 2^−ΔΔCt^ method. The data were normalizing to the 18S rRNA reference gene (see Table 1) and then expressed relative to the respective control group.

DNA Synthesis Assay: Duodenal explants (~50 mg) from 1-day-old chicks were trypsinized to dissociate cells being treated with trypsin (0.025% trypsin and ethylenediaminetetraacetic acid (EDTA) (0.5 mmol L^−1^)) at 38 °C cells for 30 min. Cells were incubated in a 24-well plate at 38 °C (5% CO_2_) with approximately 50,000 cells per well cultured in 1 mL of Eagle’s medium supplemented with bovine serum albumin (20%), glutamine (2 mM), insulin (0.6 U ml^−1^) and antibiotics (1%). Cells were pretreated with Met-enkephalin (10 nM) for 2 h; then, IGF-1 (10 nM) was added, and cells were cultured for 48 h. H^3^ thymidine (Methyl-^3^H) [Perkin Elmer, USA] (500,000 cpm) was then added to each well, and cells were incubated for an additional 24 h. The cell culture was terminated via centrifugation (10 min at 1000 rpm and 4 °C). The incorporation of H^3^-thymidine was determined in beta-counter (Beckmann).

Reagents Used in the Research: The following reagents were employed: Eagle’s medium (Biomed, Lublin, Poland), BSA and antibiotic–antimycotic solution (AAS) (Merck KGaA, Darmstadt, Germany), StayRNA (A&A Biotechnology, Gdynia, Poland), TRI reagent (MRC Inc., Cincinnati, OH, USA), High-Capacity cDNA Reverse Transcription Kit (Thermo Fisher Scientific, Waltham, MA, USA), primers (IBB PAN, Warsaw, Poland) and 5× HOT FIREPol EvaGreen qPCR Mix Plus (ROX) (Solis BioDyne, Tartu, Estonia). Other reagents were purchased from Chempur (Piekary Slaskie, Poland), Warchem (Marki, Poland) and Sigma-Aldrich (St. Louis, MO, USA).

Statistical Analysis: Data on the effects of the two cholinergic antagonists were analyzed via two-way analysis of variance (2-way ANOVA) in each of the two phases of the study. Means from ANOVA analyses were separated using Tukey’s honestly significant difference test (HSD) with *p* < 0.05 considered as significant. Effects of the opioid antagonist, naltrexone, were analyzed using Student’s *t*-test. Relationships between and among the release of Met-enkephalin, SRIF and IGF-1 together with PENK expression were analyzed via linear regression with *p* < 0.05 considered as significant. Data on Met-enkephalin and IGF-1 effects on DNA synthesis in duodenal tissue were also analyzed via 2-way ANOVA with means separated using Tukey’s HSD.

## 3. Results

### 3.1. Release of Met-Enkephalin

**Figure 1:**
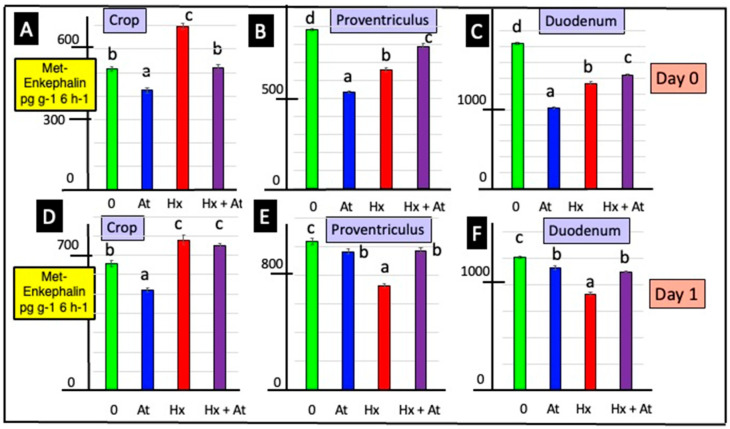
Effects of atropine and/or hexamethonium on the in vitro release of Met-enkephalin from explants of the crop, proventriculus and duodenum from newly hatched chicks. [For a, b, c and d, different lower-case letters indicate differences *p* < 0.001 for the release of Met-enkephalin from the explants of the crop (**A**,**D**), proventriculus (**B**,**E**) and duodenum (**C**,**F**) tissue from 0- (**A**–**C**) and 1- (**D**–**F**) day-old chicks].

In the presence of atropine, the release of Met-enkephalin from GI tract explants was depressed [from the crop: day 0–17.8% and day 1–20.7%; proventriculus: day 0–39.2% and day 1–6.9%; duodenum: day 0–44.4% and day 1–7.8%] (Figure 1 and Table S1).

Release of Met-enkephalin from crop explants was increased in crop explants incubated in the presence of hexamethonium [day 0–35.3% and day 1–18.5%] (according to two-way ANOVA) (Figure 1 and Table S1). In contrast, release of Met-enkephalin from proventriculus and duodenal explants was decreased (according to two-way ANOVA) in the presence of hexamethonium [proventriculus: day 0–25.1% and day 1–29.7%; duodenum: day 0–44.4% and day 1–27.4%] (Figure 1 and Table S1).

**Figure 2:**
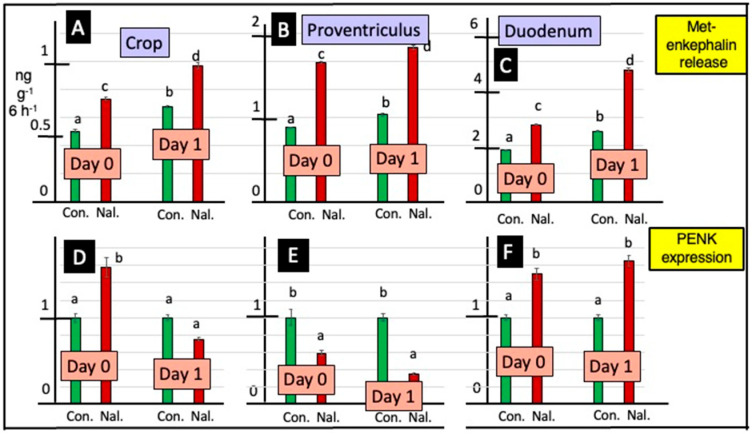
Effect of naltrexone (Nal) on Met-enkephalin release from and PENK expression in explants of the crop, proventriculus and duodenum in vitro. [con.—control; for a, b, c and d; different lower-case letters indicate difference *p* < 0.001 for release of Met-enkephalin from explants of crop (**A**), proventriculus (**B**) and duodenum (**C**) tissue from young chicks; PENK expression in explants of crop (**D**), proventriculus (**E**) and duodenum (**F**) tissue from young chicks; PENK expression is shown relative to the controls (as 1.0)].

In the presence of naltrexone, there was increased (*p* < 0.001) release of Met-enkephalin from explants (according to Student’s *t*-test), irrespective of whether from the crop, proventriculus or duodenum or tissues from day 0 or day 1 chicks (Figure 2). There was greater (*p* < 0.001) release of Met-enkephalin from duodenal explants compared to that from proventriculus, and in turn, it was greater than that from crop explants (Figure 2).

### 3.2. Expression of the PENK Gene

**Figure 3:**
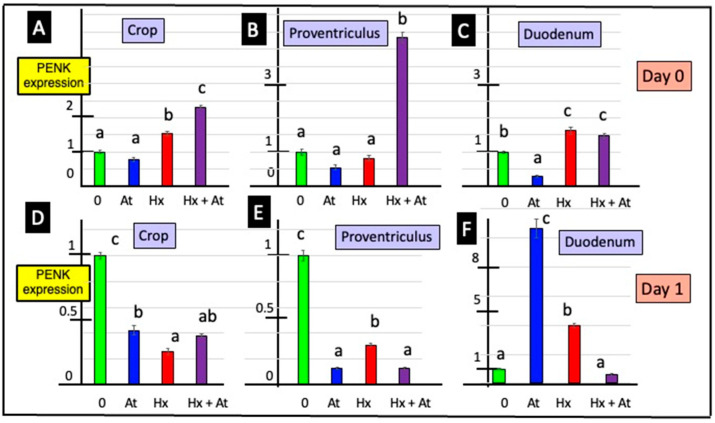
Effects of atropine (At) and/or hexamethonium (Hx) on PENK expression from explants of crop, proventriculus and duodenum from newly hatched chicks [for a, b and c, different lower-case letters indicate difference *p* < 0.001 for PENK expression in explants of crop (**A**,**D**), proventriculus (**B**,**E**) and duodenum (**C**,**F**) tissue from 0- (**A**–**C**) and 1- (**D**–**F**) day-old chicks]. PENK expression is shown relative to the controls (1.0).

Two-way ANOVA revealed effects of incubating GI explants with cholinergic antagonists on PENK expression. Expression of PENK was depressed in crop tissue explants incubated with atropine, irrespective of whether tissue was from day 0 or day 1 chicks (Figure 3 and Table S2). Moreover, PENK expression was either decreased (day 1) or tended to be decreased (day 0) in proventriculus explants incubated with atropine (Figure 3 and Table S2). The expression of PENK was increased in duodenal tissue incubated with hexamethonium, irrespective of whether the tissue was from day 0 or day 1 chicks (Figure 3 and Table S2).

There were effects of incubating GI explants with the opioid antagonist naltrexone on PENK expression, with increased expression (by Student’s *t*-test) with explants from the crop (day 0), proventriculus (day 0) and duodenum (days 0 and 1) (Figure 2).

### 3.3. Release of SRIF

**Figure 4:**
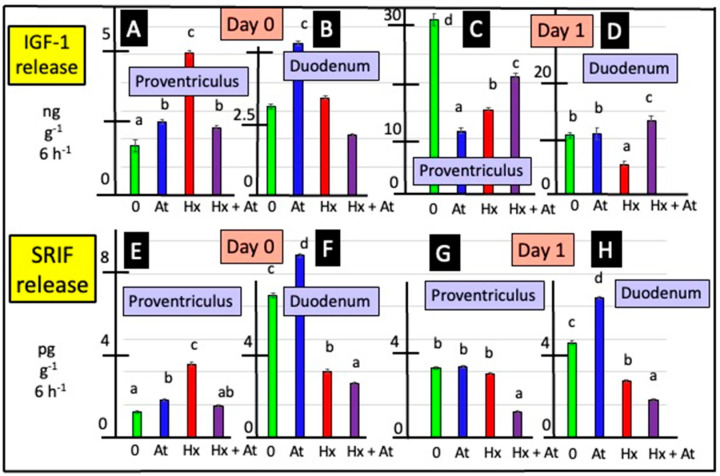
Effects of atropine (At) and/or hexamethonium (Hx) on in vitro release of SRIF and IGF-1 from explants of proventriculus and duodenum from newly hatched chicks. [For a, b, c and d, different lower-case letters indicate difference *p* < 0.001 for release of IGF-1 from explants of proventriculus (**A**,**C**) and duodenum (**B**,**D**) tissue and for release of SRIF from explants of proventriculus (**E**,**G**) and duodenum (**F**,**H**) tissue from young chicks].

**Figure 5:**
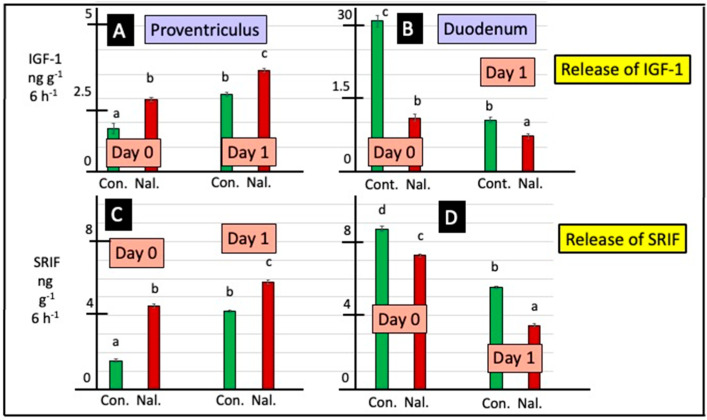
Effect of naltrexone on IGF-1 and SRIF release from explants of the proventriculus and duodenum in vitro. [For a, b, c and d, different lower-case letters indicate difference *p* < 0.001 for the release of IGF-1 from explants of proventriculus (**A**) and duodenum (**B**) tissue and for the release of SRIF from explants of proventriculus (**C**) and duodenum (**D**) tissue from young chicks].

Cholinergic antagonists influenced release of SRIF from explants of the proventriculus either from day 0 or day 1 chicks (according to two-way ANOVA) (Figure 4 and Table S3). In vitro release of SRIF from proventriculus explants of day 0 chicks was increased (*p* < 0.0001) in the presence of atropine (by 47.4%) and hexamethonium (by 2.87-fold) (Figure 4 and Table S3). Release of SRIF from duodenal explants was increased in the presence of atropine (by 27.9% in day 0 chicks and by 46.9% in day 1 chicks) (Figure 4 and Table S3). In contrast, the release of SRIF from duodenal explants was decreased in the presence of hexamethonium (by 47.0% in day 0 chicks and by 40.3% in day 1 chicks) (Figure 4 and Table S3). There was increased (*p* < 0.001) in vitro release of SRIF from explants of the proventriculus incubated with naltrexone (Figure 5). In the presence of naltrexone, there was decreased (*p* < 0.001) release of SRIF from duodenal explants in vitro (Figure 5).

### 3.4. Release of IGF-1

Release of IGF-1 in vitro was increased (*p* < 0.05) in proventriculus explants incubated in the presence of atropine (according to two-way ANOVA) (Figure 4 and Table S4). The increases were, respectively, 47.7% for tissue from day 0 and 70.9% for tissue from day 1 chicks. Conversely, tin vitro release of IGF-1 was decreased (*p* < 0.05) in duodenum explants incubated in the presence of the muscarinic antagonist, atropine (Figure 4, Table S4), with the decreases being 52.3% (day 0) and 48.1% (day 1). Moreover, there was increased (*p* < 0.05) release of IGF-1 from explants of the proventriculus (from day 0 chicks) and duodenum (day 1) incubated in the presence of hexamethonium (Figure 4 and Table S4).

Release of IGF-1 was increased (*p* < 0.001) from explants of proventriculus incubated with naltrexone in vitro (Figure 5). In contrast, release of IGF-1 from duodenal explants was decreased (*p* < 0.001) in the presence of naltrexone in vitro (Figure 5 and Table S4).

### 3.5. Relationships Between Release of Met-Enkephalin, IGF-1 and SRIF

Table 2 summarizes relationships between release of Met-enkephalin, IGF-1 and SRIF. There were strong or relatively strong relationships (adjusted R^2^ > 0.38) between the rates of release of IGF-1 and SRIF from proventriculus explants but not duodenum explants (from both day 0 and day 1 chicks). Moreover, there was a relationship (*p* < 0.001) between release of Met-enkephalin and IGF-1 from explants from the duodenum but not the proventriculus (from both day 0 and day 1 chicks) (Table 2).

### 3.6. Effects of Met-Enkephalin and/or IGF-1 on DNA Synthesis (Cell Proliferation) in Duodenal Cells

**Figure 6:**
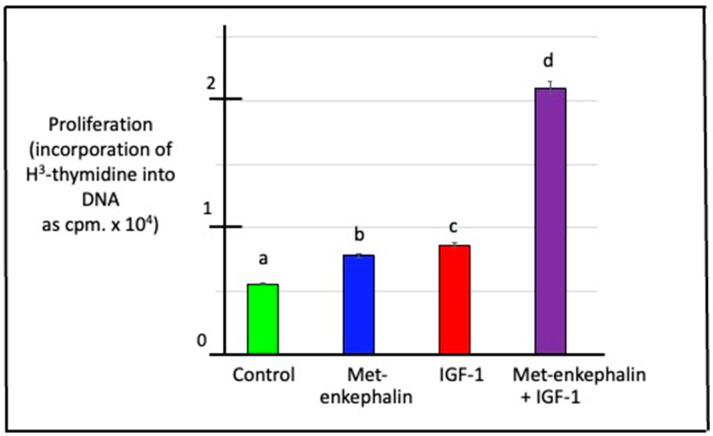
Effect of IGF-1 and/or Met-enkephalin on the proliferation of duodenal cells in explants incubated in vitro. [For a, b, c and d, different letters indicate difference *p* < 0.001].

There was increased (*p* < 0.001) proliferation of chick duodenal cells in the presence of either Met-enkephalin or IGF-1 (Met-enkephalin *p* = 3.99 × 10^−15^ and IGF-1 *p* = 7.47 × 10^−16^ according to two-way ANOVA) (Figure 6). Moreover, these peptides exerted a synergistic effect on the proliferation of chick duodenal cells (interaction between Met-enkephalin and IGF-1, *p* = 1.15 × 10^−12^) (Figure 6).

## 4. Discussion

This is the first report that the crop is a site of both the release and synthesis of a neuropeptide, namely Met-enkephalin (Figure 1 and Figure 2 and Tables S1 and S2). There was greater release of Met-enkephalin from the proventriculus than crop tissue (Table S1 and Figure 1). Moreover, there was both greater release of Met-enkephalin from and expression of PENK in the duodenum than in the proventriculus (Figure 1 and Tables S1 and S2). Similarly, there was greater in vitro release of SRIF and IGF-1 with explants from the duodenal than from the proventriculus (Tables S3 and S4).

In the present studies, SRIF release was influenced by the presence of cholinergic receptors antagonists. In the mammalian stomach, SRIF is produced by both the D endocrine cells and the enteric nervous system in both the submucosal plexus and the myenteric plexus. However, SRIF is produced by only the enteric nervous system in the small intestine (pigs: [28]; also reviewed: [29,30]). There were disparate effects of cholinergic antagonists on SRIF release from proventriculus or duodenal explants; the increased release of SRIF from proventriculus explants and the decreased release of SRIF from duodenal explants in the presence of hexamethonium were observed (Figure 4). What is not clear is whether the different sites of SRIF synthesis influence the mechanisms of control. In vitro SRIF release was increased when duodenal explants were incubated with atropine (Figure 4). The present results are consistent with reports of cholinergic effects on SRIF release in rodents [46]. The muscarinic antagonist, atropine, partially overcame the decrease in SRIF release following either electrical stimulation or the infusion of peptone solutions in vascularly perfused rat stomachs [26,47] or gastric distention (rats: [25,27]). Moreover, the distention of the stomach activates cholinergic reflexes [48]. However, the effect of hexamethonium in depressing SRIF release from duodenal explants is novel.

IGF-1 exerts an autocrine role that stimulates the growth of multiple tissues. There is evidence, albeit limited, for the intestinal synthesis and/or release of IGF-1. The release of IGF-1 from small intestine cells occurs during the regenerative phase of crypt repair after radiation treatment [49]. Intestinal production of IGF-1 is related to nutrition and intestinal hormones (reviewed: [50]). For instance, fasting reduces jejunal IGF-I mRNA with the effect being overcome with re-feeding [51]. The present report on the effects of cholinergic antagonists in IGF-1 release is novel and supports the neural control of intestinal IGF-1 release.

In duodenal explants, there were relationships between the release of Met-enkephalin and that of IGF-1 (Table 2). Similarly, IGF-I increased the concentration of proenkephalin-derived enkephalin motif -containing peptides in bovine adrenal chromaffin cells [52]. The following question arises: is it possible that IGF-1 and Met-enkephalin interact via the activation of similar/identical signaling pathways in the chick duodenum? It was noted that IGF-1 decreases protein levels of brain-derived neurotrophic factor (BDNF) after 24 h; BDNF is a neurotropin that inhibits the proliferation of retinal cells [53]. On the other hand, endogenous Met-enkephalin upregulated BDNF mRNA through δ- and μ-opioid receptors in rats [54]. In the present experiment, Met-enkephalin and IGF-1 increased the rate of proliferation in vitro of duodenal, presumably epithelial, cells (Figure 6). This suggests that the potentiated effect may be due to the activation of separate pathways.

There are other instances of relationships between enteric peptides. In rats receiving enteric nutrition or glucagon-like peptides, ileal expression of IGF-1 correlated with concentrations of gut hormones (proglucagon) or their receptors (glucagon-like peptide receptor 2R) [55]. The relationship between the in vitro release of SRIF and that of IGF-1 in the proventriculus explants incubated in the presence or absence of cholinergic antagonists is intriguing. This may be explicable by their production in the same cells and needs investigation.

## 5. Conclusions

Met-enkephalin was released in vitro from the crop, proventriculus and duodenum of young chicks. Similarly, PENK is expressed in the crop, proventriculus and duodenum of young chicks. This is the first report of a neuropeptide present in the crop. In addition, there was the release of SRIF and IGF-1 from explants of the proventriculus and duodenum. In the presence of cholinergic and opioid antagonists, there were shifts in the in vitro release of Met-enkephalin, SRIF and IGF-1 and in the expression of PENK in different GI regions. These are consistent with both cholinergic and endogenous opioid control of the release of Met-enkephalin, SRIF and IGF-1 together with PENK expression. This is the first report of nervous control of IGF-1 release.

## Figures and Tables

**Table 1:** Primers for Met-Enkephalin and 18S rRNA used in the real-time PCR and amplified fragment size (bp).

Primer	F/R	Gene Sequence	Fragment Size (bp)
pPENK	F	5′-CAGCTCTTTGGCTTCATCT-3′	102
	R	5′-AGAGGCCAATGGAAGTGAGA-3′	
18S rRNA	F	5′-CTTTGGTCGCTCGCTCCTC-3′	115
	R	5′-CTGACCGGGTTGGTTTTGAT-3′	

**Table 2:** Relationships between release of Met-enkephalin and that of IGF-1 and SRIF from explants of GI tracts from newly hatched chicks.

	Adjusted R^2^	*p*-Value
Proventriculus		
Day 0		
Met-enkephalin vs. IGF-1	0.138	0.0595
Met-enkephalin vs. SRIF	0.158	0.0465
SRIF vs. IGF-1	0.897	1.52 × 10^−10^
Day 1		
Met-enkephalin vs. IGF-1	−0.055	0.948
Met-enkephalin vs. SRIF	−0.044	0.659
SRIF vs. IGF-1	0.488	0.00037
Duodenum		
Day 0		
Met-enkephalin vs. IGF-1	0.899	1.34 × 10^−10^
Met-enkephalin vs. SRIF	−0.0012	0.390
SRIF vs. IGF-1	−0.051	0.783
Day 1		
Met-enkephalin vs. IGF-1	0.388	0.00199
Met-enkephalin vs. SRIF	0.166	0.0424
SRIF vs. IGF-1	−0.049	0.734

## Data Availability

Data are available by request to the authors.

## Supplementary Materials

The following supporting information can be downloaded at https://www.mdpi.com/article/10.3390/ani15121702/s1, Table S1. In vitro effects on atropine and/or hexamethonium on Met-enkephalin release from explants of the crop, proventriculus and duodenum from newly hatched chicks; Table S2. In vitro effects of atropine and/or hexamethonium on PENK expression in explants of the crop, proventriculus and duodenum from newly hatched chicks; Table S3. In vitro effects of atropine and/or hexamethonium on SRIF release from explants of the proventriculus and duodenum from newly hatched chicks; Table S4. In vitro effects of atropine and/or hexamethonium on IGF-1 release from explants of the proventriculus and duodenum from newly hatched chicks.
