# Supplementary material for: Effects of Cholinergic and Opioid Antagonists on In Vitro Release of Met-Enkephalin, Somatostatin and Insulin-like Growth Factor-1 by and PENK Expression in Crop, Proventriculus and Duodenum of Newly Hatched Chickens
Source: Animals (Basel). 2025 Jun 9;15(12):1702. doi: 10.3390/ani15121702 (PMC12189355; doi:10.3390/ani15121702)
Supplement: Supplementary file 1 [file animals-15-01702-s001.zip › animals-3484093-supplementary.pdf]

Supplement Table S1. *In vitro* Effects on atropine and/or hexamethonium on Met-enkephalin release from explants of crop, proventriculus and duodenum from newly hatched chicks.

Group/tissue	Release of Met-enkephalin pg g^{-1} 6 h^{-1}	
	day 0	day 1
**Crop**		
Control	516 ± 7.58[b]	658 ± 15.8[b]
Atropine	424 ± 12.4[a]	522 ± 9.84[a]
Hexamethonium	698 ± 13.6[c]	780 ± 26.8[c]
Atropine + hexamethonium	519 ± 14.5[b]	748 ± 13.6[c]
**2-way ANOVA**	*P* =	*P* =
Atropine	$6.31E^{-6}$	0.000453
Hexamethonium	$8.99E^{-6}$	$4.28E^{-7}$
Interaction	0.945	0.0125
**Proventriculus**		
Control	885 ± 6.18[d]	1034 ± 19.9[a]
Atropine	538 ± 6.20[a]	963 ± 19.5[b]
Hexamethonium	663 ± 11.2[b]	727 ± 14.0[c]
Atropine+hexamethonium	792 ± 13.3[c]	971 ± 19.2[b]
**2-way ANOVA**	*P* =	*P* =
Atropine	$5.46E^{-9}$	0.000232
Hexamethonium	0.117	$4.30E^{-7}$
Interaction	$4.01E^{-14}$	$2.27E^{-7}$
**Duodenum**		
Control	1833 ± 16.7[d]	2521 ± 18.2[c]
Atropine	1019 ± 17.1[a]	2324 ± 31.9[b]
Hexamethonium	1331 ± 18.5[b]	1830 ± 23.4[a]
Atropine+hexamethonium	1439 ± 14.4[c]	2253 ± 16.1[b]
**2-way ANOVA**	*P* =	*P* =
Atropine	$4.28E^{-13}$	0.000170
Hexamethonium	0.0273	$1.90E^{-11}$
Interaction	$6.72E^{-15}$	$4.19E^{-10}$

[a, b, c, d] Different superscript letters indicate difference between treatments $P < 0.05$

Supplement Table S2. *In vitro* effects of atropine and/or hexamethonium on PENK expression in explants of crop, proventriculus and duodenum from newly hatched chicks.

	Expression of PENK $\pm$ (n = 3) SEM pg mg^{-1}	
	**Day 0**	**Day 1**
**Crop**		
Control	1.43 ± 0.066^{a}	5.42 ± 0.148^{a}
Atropine	1.13 ± 0.081^{a}	2.29 ± 0.196^{b}
Hexamethonium	2.21 ± 0.069^{b}	1.40 ± 0.140^{c}
Atropine + Hexamethonium	3.31 ± 0.071^{c}	2.07 ± 0.074^{bc}
**2-way ANOVA**	*P* =	*P* =
Atropine	0.000547	0.0000314
Hexamethonium	3.35E^{-8}	5.06E^{-7}
Interaction	9.94E^{-6}	0.00000118
**Proventriculus**		
Control	1.31 ± 0.116^{a}	7.38 ± 0.283^{a}
Atropine	0.73 ± 0.087^{a}	0.94 ± 0.056^{c}
Hexamethonium	1.08 ± 0.101^{a}	2.28 ± 0.106^{b}
Atropine + Hexamethonium	5.70 ± 0.200^{b}	0.96 ± 0.075^{c}
**2-way ANOVA**	*P* =	*P* =
Atropine	3.60E^{-7}	8.10E^{-9}
Hexamethonium	1.02E^{-7}	2.25E^{-7}
Interaction	4.95E^{-8}	2.12E^{-7}
**Duodenum**		
Control	2.85 ± 0.085^{b}	2.00 ± 0.074^{a}
Atropine	0.84 ± 0.079^{c}	21.3 ± 1.25^{c}
Hexamethonium	4.63 ± 0.333^{a}	7.99 ± 0.261^{b}
Atropine + Hexamethonium	4.19 ± 0.244^{a}	1.18 ± 0.061^{a}
**2-way ANOVA**	*P* =	*P* =
Atropine	0.000456	1.01E^{-5}
Hexamethonium	2.19E^{-6}	4.03E^{-6}
Interaction	0.00643	3.48E^{-8}

$^{a, b, c, d}$ Different superscript letters indicate difference between treatments $P < 0.05$

Supplement Table S3. *In vitro* effects of atropine and/or hexamethonium on SRIF release from explants of proventriculus and duodenum from newly hatched chicks.

Group/tissue	**Release of SRIF** Mean ± (n = 5) SEM pg mg^{-1}	
	day 0	day 1
**Proventriculus**		
Control	1.56 ± 0.093^{a}	4.24 ± 0.068^{b}
Atropine	2.30 ± 0.084^{b}	4.30 ± 0.071^{b}
Hexamethonium	4.48 ± 0.128^{c}	3.88 ± 0.073^{b}
Atropine +hexamethonium	1.90 ± 0.071^{ab}	1.58 ± 0.086^{a}
2-way ANOVA	*P* =	*P* =
Atropine	$5.08E^{-8}$	$7.90E^{-11}$
Hexamethonium	$5.71E^{-10}$	$6.16E^{-11}$
Interaction	$9.16E^{-12}$	$3.60E^{-11}$
**Duodenum**		
Control	8.68 ± 0.131^{c}	5.80 ± 0.100^{c}
Atropine	11.1 ± 0.114^{d}	8.52 ± 0.086^{d}
Hexamethonium	4.06 ± 0.150^{b}	3.46 ± 0.093^{b}
Atropine +hexamethonium	3.32 ± 0.086^{a}	2.32 ± 0.073^{a}
2-way ANOVA	*P* =	*P* =
Atropine	$4.00E^{-6}$	$1.32E^{-7}$
Hexamethonium	$4.56E^{-19}$	$9.45E^{-19}$
Interaction	$7.52E^{-10}$	$2.55E^{-13}$

[a, b, c, d] Different superscript letters indicate difference between treatments $P < 0.05$

Supplement Table S4. *In vitro* effects of atropine and/or hexamethonium on IGF-1 release from explants of proventriculus and duodenum from newly hatched chicks.

Group/tissue	Release of IGF-1 Mean ng mg^{-1} 6 h^{-1}	
	day 0	day 1
**Proventriculus**		
Control	1.76 ± 0.211[a]	3.16 ± 0.081[b]
Atropine	2.60 ± 0.071[b]	5.40 ± 0.071[c]
Hexamethonium	5.04 ± 0.112[c]	3.46 ± 0.068[b]
Atropine + hexamethonium	2.40 ± 0.089[b]	2.12 ± 0.073[a]
	P =	*P* =
Atropine	$4.31E^{-6}$	$1.47E^{-5}$
Hexamethonium	$3.25E^{-9}$	$7.74E^{-13}$
Interaction	$5.50E^{-10}$	$4.49E^{-14}$
Group/tissue		
**Duodenum**		
Control	31.2 ± 1.14[d]	10.6 ± 0.51[b]
Atropine	11.4 ± 0.51[a]	11.0 ± 1.05[a]
Hexamethonium	15.2 ± 0.37[b]	5.50 ± 0.51[a]
Atropine + hexamethonium	21.0 ± 0.71[c]	13.2 ± 0.80[c]
	P =	*P* =
Atropine	$7.42E^{-8}$	$5.29E^{-5}$
Hexamethonium	0.000707	*0.0633*
Interaction	$1.03E^{-11}$	0.000153

[a, b, c, d] Different superscript letters indicate difference between treatments $P < 0.05$
